# Compositional and Functional Characterization of Glycolipid Biosurfactants from an Olive-Derived *Lactiplantibacillus plantarum*

**DOI:** 10.4014/jmb.2606.06027

**Published:** 2026-08-10

**Authors:** Jong Woo Hyeon, Min Hui Jeon, Jun-Tae Bae, Man-Young Jung

**Affiliations:** 1 Interdisciplinary Graduate Program in Advanced Convergence Technology and Science, Jeju National University, Jeju 63243, Republic of Korea; 2 R&D Center, J2KBIO, Cheongju 28104, Republic of Korea; 3 Department of Biology Education, Jeju National University, Jeju 63243, Republic of Korea; 4 Bio-Health Materials Core-Facility Center, Jeju National University, Jeju 63243, Republic of Korea; 5 Jeju Microbiome Center, Jeju National University, Jeju 63243, Republic of Korea

**Keywords:** *Lactiplantibacillus plantarum*, Biosurfactant, Glycolipids, Phenyllactic acid, Antifungal activity, Anti-inflammatory effect

## Abstract

Biosurfactants from lactic acid bacteria are attractive as safe, multifunctional ingredients for food and cosmetic applications, yet many remain only partially characterized. An olive-derived strain was identified as *Lactiplantibacillus plantarum* J2K-1229 by 16S rRNA gene and genome-based phylogeny. Grown in rapeseed oil-containing medium, it secreted extracellular material with surface/interfacial activity, and its cell-free supernatant emulsified diverse plant oils (emulsification index after 24 h, 26.67−41.33%). A purified biosurfactant fraction gave carbohydrate- and lipid-positive, ninhydrin-negative thin-layer chromatography and Fourier transform infrared bands of a hydroxylated, ester- and carboxylic acid-containing lipid. Gas chromatography, NMR, and HPAEC–PAD indicated a complex, lipid-dominant mixture with major oleic and palmitic acids, NMR signals consistent with co-extracted phenyllactic acid-type metabolites, and fructose as the only detected monosaccharide. Genome mining revealed glucosyltransferases for glucose-based glycolipids but no fructosyltransferase, suggesting that the detected fructose may represent co-extracted free fructose, possibly released as a glucansucrase by-product. The preparation inhibited *Candida albicans* in disk diffusion assays and was non-cytotoxic to RAW 264.7 macrophages and HaCaT keratinocytes up to 200 ppm. In lipopolysaccharide-stimulated macrophages it suppressed nitric oxide production (88.07% at 200 ppm) and inducible nitric oxide synthase expression, and in cytokine-stimulated keratinocytes it reduced TARC, MDC, and RANTES expression. Overall, this olive-derived *L. plantarum* produces a multicomponent, glycolipid-containing biosurfactant preparation with emulsifying, antifungal, and anti-inflammatory activities suited to cosmetic and personal-care applications.

## Introduction

Surfactants are indispensable in diverse industrial sectors, including food processing, agriculture, pharmaceuticals, and personal care, because amphiphilic molecules can reduce surface and interfacial tension and promote emulsification and wetting. However, extensive use of petroleum-derived synthetic surfactants has raised concerns related to environmental persistence, ecotoxicity, and potential health issues, thereby accelerating the search for safer and more sustainable surface-active alternatives [1-3]. In this context, biosurfactants, surface-active compounds produced by microorganisms, have attracted attention due to their biodegradability, lower toxicity, and functionality under a broad range of conditions, often accompanied by additional bioactivities such as antimicrobial and antiadhesive effects [4-6].

A major barrier to wider application of biosurfactants is that high-performing producers are frequently opportunistic or pathogenic microorganisms, complicating regulatory acceptance for food and cosmetic uses. Accordingly, biosurfactants from lactic acid bacteria (LAB) have emerged as attractive candidates because LAB have a long history of safe use in fermented foods and many strains are considered suitable for applications requiring high safety margins [6]. A growing body of work indicates that LAB can produce surface-active compounds that are cell-associated or secreted, and that these materials can inhibit pathogen adhesion and biofilm formation, providing opportunities for biopreservation and hygiene-related applications [7, 8]. Within LAB, *Lactiplantibacillus plantarum* (formerly *Lactobacillus plantarum*) is a metabolically versatile species frequently isolated from plant-associated environments and fermented plant matrices, making it a promising source for discovering bioactive metabolites from agro-food ecosystems [9]. Notably, olive fermentations and related brines represent a niche environment enriched in LAB adapted to salt, acidic pH, and plant-derived substrates; *L. plantarum* strains from olive-related sources have been reported to produce biosurfactant-like molecules with emulsifying and antimicrobial properties [10]. Moreover, because the olive matrix is lipid-rich and imposes combined osmotic and acidic stress, this niche may favor strains with active membrane-lipid remodeling and extracellular surface-active metabolism, strengthening the rationale for targeting olive-derived *L. plantarum*. Thus, olive-associated LAB represent a relevant but still underexplored source of safe surface-active and bioactive preparations from plant-fermentation ecosystems.

Despite increasing reports of LAB-derived biosurfactants, progress toward application is hampered by limited compositional resolution and standardization. Many studies describe LAB biosurfactants using bulk composition, such as carbohydrate/protein/lipid ratios, and functional assays, while full molecular structures are only rarely resolved, partly because these preparations are frequently complex mixtures rather than a single compound [6, 11]. This limitation is critical because the overall chemical composition of a biosurfactant preparation and the molecular structure of its individual active components determine its physicochemical performance and bioactivity. Incomplete characterization of either aspect restricts reproducibility, comparability across studies, and rational optimization for target applications [11]. For *L. plantarum*, detailed structural work on cell-envelope glycolipids has demonstrated that this species produces multiple glycosyl-diacylglycerols (*e.g.*, glucose/galactose-containing glycolipids), underscoring the inherent diversity of glycolipid architectures in this taxon [12]. Beyond these membrane glycolipids, strains of this species also produce exopolysaccharides [13] and secrete antifungal phenyllactic acids, including phenyllactic and p-hydroxyphenyllactic acid [14, 15]. Consequently, a single solvent extraction can co-recover several surface-active and bioactive species. Interpreting such preparations therefore benefits from pairing compositional analysis with genome-based inference of biosynthetic capacity, which indicates the lipid, glycolipid, and metabolite classes that a given strain is genetically equipped to synthesize. Therefore, resolving the composition, function, and genomic basis of glycolipid biosurfactant preparations from *L. plantarum* strains associated with plant fermentations could expand the repertoire of safe, bio-based surfactants and clarify structure–function relationships.

In the present study, we isolated a biosurfactant-producing LAB strain from olives and identified it as *L. plantarum* J2K-1229 by 16S rRNA gene and whole-genome phylogeny. An extracellular surface-active fraction produced in rapeseed oil-containing medium was fractionated and characterized using thin-layer chromatography (TLC), Fourier transform infrared (FT–IR) spectroscopy, gas chromatography of fatty acid methyl esters (GC–FAME), high-performance anion-exchange chromatography with pulsed amperometric detection (HPAEC–PAD) and one-dimensional ^1^H and ^13^C nuclear magnetic resonance (NMR) spectroscopy. Genome mining was used to interpret the chemical features in relation to fatty acid, glycolipid, exopolysaccharide, and phenyllactic acid-related metabolism. Because LAB-derived biosurfactant preparations can be chemically complex, compositional analysis, genome-based interpretation, and functional assays were combined to distinguish the properties of the preparation from those of a single defined molecule. The preparation was further evaluated for emulsification, antimicrobial, cytotoxicity, and anti-inflammatory activities. Overall, this study aimed to clarify the properties and limitations of a multicomponent glycolipid-containing biosurfactant preparation from olive-derived *L. plantarum*.

## Materials and Methods

### Isolation and Enrichment of Biosurfactant-Producing LAB

Olives were ground using a sterilized mortar and pestle, and suspended in phosphate-buffered saline (PBS; 137 mM NaCl, 2.7 mM KCl, 10 mM Na_2_HPO_4_, 2 mM KH_2_PO_4_, pH 7.2). The suspension was inoculated into customized De Man, Rogosa and Sharpe (cMRS) broth (1% peptone, 1.5% yeast extract, 0.01% MgSO_4_∙7H_2_O, 0.2% K_2_HPO_4_, 0.2% ammonium citrate tribasic, 0.5% sodium acetate, 0.005% MnSO_4_∙H_2_O, 10% rapeseed oil (w/v)) and incubated aerobically at 35°C with shaking at 150 rpm for 7 days. This glucose- and Tween-free, rapeseed-oil-containing medium was used to enrich LAB candidates adapted to lipid-rich conditions and potentially capable of producing extracellular surface/interfacial-active compounds. After incubation, the culture was subcultured in fresh cMRS broth; this procedure was repeated three times in total. The third culture was serially diluted to 10^−6^ in PBS and 0.1 mL aliquots of the 10^−4^–10^−6^ dilutions were spread onto cMRS agar and incubated at 35°C for 3 days. Colonies formed on cMRS agar were screened based on colony morphology and size, and stored in cMRS broth containing 15% (v/v) glycerol at -80°C for long-term preservation.

### Oil-Spreading Assay

Strains isolated from olives were inoculated into 5 mL of cMRS broth and incubated at 35°C with shaking at 150 rpm for 3 days. After cultivation, each culture was centrifuged at 14,000 rpm for 10 min, and only the aqueous cell-free supernatant was carefully harvested after excluding the floating oil layer. This sample was used for the oil-spreading assay to evaluate extracellular surface/interfacial activity independently of residual oil. The cell pellets were washed twice with distilled water and resuspended in 5 mL of PBS. The suspensions were incubated on a magnetic stirrer for 4 h at room temperature with gentle stirring to release cell-bound biosurfactants.

The oil-spreading assay was performed as described by Joe *et al*. (2019) [16]. Briefly, 50 mL of distilled water was added to a Petri dish, and 20 μL of rapeseed oil was gently layered onto the water surface. Subsequently, 2 μL of the oil-free supernatant or the PBS cell extract was carefully dropped onto the oil surface, and the formation of a clear zone was observed.

### Phylogenetic Classification based on 16S rRNA Gene Sequences

The 16S rRNA gene of the strain selected by the oil-spreading assay was amplified by polymerase chain reaction (PCR) using the universal primers F1 (5′-AGAGTTTGATCMTGGCTCAG-3′) and R13 (5′-TACGGYTACCTTGTTACGACTT-3′) [17]. The PCR amplicon was sequenced using the universal primers 340F (5′-CCTACGGGAGGCAGCAG-3′), 518R (5′-ATTACCGCGGCTGCTGG-3′), and 805F (5′-GATTAG ATACCCTGGTAGTC-3′) to obtain complete 16S rRNA gene sequences [17]. The resulting sequence was compared with those of closely related species using the nucleotide similarity-based EzBioCloud server [18].

The 16S rRNA gene sequences of the strain and closely related type strains were aligned using ClustalW program in MEGA X. Phylogenetic trees were reconstructed using the neighbor-joining (NJ), maximum-parsimony (MP), and maximum-likelihood (ML) algorithms in MEGA X, with bootstrap analysis based on 1,000 replicates [19]. The evolutionary distances of the NJ algorithm were computed using the Kimura 2-parameter model [20], the MP tree was obtained using the Subtree-Pruning-Regrafting (SPR) method [20], and the Tamura Nei model was applied in the ML tree using the Nearest-Neighbor-Interchange (NNI) method [21].

### Whole-Genome Sequencing, Phylogenomic Analysis, and Functional Genome Mining

Genomic DNA of strain J2K-1229 was extracted, and whole-genome sequencing was performed using the PacBio Revio platform (Macrogen, Republic of Korea). The quality of raw HiFi reads was assessed using SeqKit v2.1.0 [22] and adapter sequences were removed using HiFiAdapterFilt v3.0.0 [22]. The PacBio BAM file was additionally checked using SAMtools v1.13 [24]. The resulting reads were *de novo* assembled using Flye v2.9.5-b1801 with built-in Flye polisher [24]. Genome quality and completeness were estimated using CheckM2 v1.1.0 [26].

Phylogenomic analysis of strain J2K-1229 and closely related *Lactiplantibacillus* spp. was conducted using the Genome Taxonomy Database Toolkit (GTDB-Tk) based on concatenated 120 phylogenetically informative markers (bac120) [27]. The resulting aligned amino acid sequences were used to reconstruct ML tree in MEGA X with bootstrap analysis based on 1,000 replicates. In addition, genome relatedness between strain J2K-1229 and type strains of closely related *Lactiplantibacillus* spp. was assessed by calculating average nucleotide identity (ANI) using FastANI v1.34 [28] and digital DNA-DNA hybridization (dDDH) using the Genome-to-Genome Distance Calculator (GGDC 3.0; https://ggdc.dsmz.de/ggdc.php) with formula 2 [29].

The assembled circular genome of strain J2K-1229 was annotated using Bakta v1.11.4 with the Bakta database v6.0 (full) [30] and eggNOG-mapper v2.1.12 with DIAMOND search strategy [31]. AMRFinderPlus v4.0.23 was used for detection of acquired antibiotic resistance and virulence genes [32]. Candidate genes related to glycolipid-type biosurfactant production and to the broader membrane-lipid and secondary-metabolite repertoire of the strain were screened from both annotations by focusing on lipase/esterase, fatty acid biosynthesis, exogenous fatty acid incorporation, glycerolipid metabolism, glycerophospholipid metabolism, glycosyltransferase functions, and exopolysaccharide/capsular polysaccharide biosynthesis, sucrose metabolism, and phenyllactic acid biosynthesis. The putative glycolipid precursor and membrane-lipid biosynthesis model was reconstructed using Kyoto Encyclopedia of Genes and Genomes (KEGG) analysis tool, KEGG Mapper [33, 34]. The KEGG orthology (KO) numbers were assigned from eggNOG-mapper, and the KO numbers were submitted to KEGG Mapper. In addition, the genomic neighborhoods of the candidate glycolipid/aminoacyl-lipid and exopolysaccharide biosynthesis gene clusters were retrieved from the Bakta annotation and rendered as scaled gene-arrow maps with in-house Python scripts to visualize their genomic organization.

### Phenotypic Characterization Using API ZYM and API 50 CHL

Phenotypic characteristics of the isolated LAB strain were evaluated using the API ZYM and API 50 CHL systems (bioMérieux, France) according to the manufacturer’s instructions. API ZYM was used to profile enzymatic activities, and API 50 CHL was used to assess carbohydrate fermentation patterns. The strips were incubated under the conditions specified in the manufacturer’s protocol, and the reactions were recorded based on the observed color changes and interpreted using the criteria provided by the manufacturer.

### Extraction of Biosurfactant Fraction from LAB

Biosurfactants were extracted from LAB as previously described [8, 35]. The biosurfactant-producing strain was inoculated into cMRS broth and incubated aerobically at 35°C with shaking at 150 rpm for 3 days. After incubation, the culture was centrifuged at 10,000 rpm for 10 min to remove bacterial cells. For biosurfactants extraction, unlike the oil-spreading assay, the non-cellular fraction including both the aqueous supernatant and the emulsified/residual oil phase, was collected to improve recovery of extracellular surface-active compounds associated with the oil-water interface or emulsion phase. The supernatant was acidified to pH 2.0 with 6 N HCl and incubated overnight at 4°C. Hexane was then added to remove residual rapeseed oil. The aqueous phase was extracted three times with ethyl acetate, and anhydrous sodium sulfate was added to the pooled organic phase to remove residual water. The dried organic phase was evaporated using a rotary evaporator to obtain crude biosurfactants. If necessary, residual oil was further removed by dissolving the crude extract in methanol and extracting with additional hexane.

### Emulsification Index (E_24_)

The emulsification index (E_24_) was determined as previously described [36, 37]. The biosurfactant-producing strain was inoculated into 5 mL of cMRS broth and incubated at 35°C with shaking at 150 rpm for 3 days. After incubation, the culture was centrifuged at 10,000 rpm for 10 min. Then, 2 mL of the cell-free supernatant after removal of the oil layer was mixed with an equal volume of each oil (jojoba, olive, avocado, argan, soybean, macadamia, evening primrose, moringa, meadowfoam seed and rapeseed oils). The mixtures were vortexed at high speed for 2 min and allowed to stand for 24 h at room temperature. E_24_ was calculated as follows:

E24 (%) = (height of the emulsion layer / total height of the mixture) × 100

### Chemical Characterization of the Purified Biosurfactant Fraction

The crude biosurfactant extract was fractionated by normal-phase chromatography using a Teledyne ACCQ Prep system HP150 equipped with a YMC-Pack SIL-HG column (30 mm × 250 mm, 10 μm). The crude extract was eluted with a gradient of hexane/ethyl acetate from 100:0 to 70:30 (v/v), and detection was performed at 214 nm. Fractions were collected and evaporated using a rotary evaporator and the resulting concentrate was confirmed to exhibit surface activity using the oil-spreading assay.

The chemical composition of the purified biosurfactant fraction was characterized using TLC, FT–IR, GC–FAME, HPAEC–PAD, and one-dimensional ^1^H and ^13^C NMR. For TLC analysis, purified biosurfactant fraction was dissolved in methanol and spotted onto a silica gel plate. After spotting, the purified biosurfactant fraction was eluted with ethyl acetate/hexane (4:1, v/v) as the mobile phase. Sugars were detected by spraying anisaldehyde-sulfuric acid-glacial acetic acid (1:2:100, v/v) [38]. Glycolipids were detected by spraying 15% (v/v) H_2_SO_4_ in ethanol [39], and lipids were detected using iodine vapor [40]. Lipopeptides were detected using ninhydrin reagent [41].

Functional groups were analyzed using a Bruker Vertex 70V FT–IR spectrometer over the wavenumber range of 600–4,000 cm^-1^. Fatty acid moieties were saponified and methylated according to the standard MIDI protocol. Fatty acid methyl esters (FAMEs) were analyzed by gas chromatography (GC–FAME; Hewlett-Packard 6890) and identified using the TSBA6 database of the Microbial Identification System [42]. The sugar moiety was determined by HPAEC–PAD using monosaccharide standards (fucose, rhamnose, arabinose, galactose, glucose, mannose, xylose, fructose). The purified biosurfactant fraction was hydrolyzed with H_2_SO_4_ and diluted with distilled water. HPAEC–PAD was performed using a Dionex ICS-5000 system (Thermo Fisher Scientific, USA) equipped with a CarboPac PA-1 column (4 mm × 250 mm). The hydrolysate was eluted with 18 mM NaOH at a flow rate of 1 mL/min.

NMR spectra were acquired on a Bruker Avance III 400 MHz spectrometer equipped with a 5 mm broadband observe (BBO) probe at the Korea Basic Science Institute (KBSI, Republic of Korea). All experiments were performed at 298 K using D_2_O as the solvent. The ^1^H NMR spectrum was recorded at 400.13 MHz using the zg30 pulse sequence with 128 scans, a spectral width of 6720 Hz, an acquisition time of 4.88 s, and a relaxation delay of 2.0 s. The ^13^C NMR spectrum was acquired at 100.62 MHz using the zgpg30 pulse sequence with broadband proton decoupling (WALTZ-16). A total of 8192 scans were collected with a spectral width of 26,042 Hz, an acquisition time of 1.26 s, and a relaxation delay of 2.0 s. All spectra were processed using Bruker TopSpin software with exponential line broadening of 1 Hz prior to Fourier transformation.

### Antimicrobial Activity

The antimicrobial activity of purified biosurfactant fraction was evaluated using the disk diffusion assay. *Staphylococcus aureus* ATCC 6538, *Escherichia coli* ATCC 8739, *Pseudomonas aeruginosa* ATCC 9027 and *Candida albicans* ATCC 10231 were used as test strains. Sterile PBS was used as a negative control. The purified biosurfactant fraction was dissolved in sterile PBS at final concentrations of 1, 2, 5, and 10% (w/v) for the test groups.

### Cell Viability Assay

The viability of RAW 264.7 cells and HaCaT cells was assessed using 3-(4,5-dimethylthiazol-2-yl)-2,5-diphenyl tetrazolium bromide (MTT) assay [43]. RAW 264.7 cells were cultured in Dulbecco's Modified Eagle’s Medium (DMEM; Sigma-Aldrich), and HaCaT cells were cultured in DMEM (1x; Welgene, Republic of Korea). Both media were supplemented with 10% fetal bovine serum (FBS; Corning) and 1% penicillin-streptomycin (Corning, USA), and cells were maintained in a humidified incubator at 37°C with 5% CO_2_. A biosurfactant solution (BS solution) was prepared by dissolving purified biosurfactant fraction in distilled water and diluted to working concentrations. Cells were seeded in a 96-well plate at a density of 1 × 10^4^ cells per well and incubated overnight at 37°C with 5% CO_2_. After incubation, cells were treated with the BS solution at various concentrations and incubated for 24 h under the same conditions. Then MTT was added to each well to a final concentration of 0.5 mg/mL and reacted at 37°C for 2 h. The supernatant was removed, and the formazan precipitate was dissolved in dimethyl sulfoxide (DMSO). Absorbance was measured at 590 nm using a microplate reader (SpectraMax ABS Plus; Molecular Devices, USA).

### Inhibition of Nitric Oxide (NO) Production

NO production was measured using the Griess reagent reaction [44]. The RAW 264.7 cells were seeded in a 96-well plate at a density of 1 × 10^4^ cells per well and incubated overnight at 37°C and 5% CO_2_. Serial dilutions of the BS solution were added at non-cytotoxic concentrations determined by the MTT assay and incubated for 2 h. After incubation, cells were stimulated with 0.5 μg/mL of lipopolysaccharide (LPS; Sigma-Aldrich) for 24 h. The cell-free supernatant was mixed with an equal volume of Griess reagent in a 96-well plate and incubated for 10 min at room temperature. Absorbance was measured at 540 nm using a microplate reader. Quercetin was used as a positive control.

### Repression of Inducible Nitric Oxide Synthase (iNOS) Expression

iNOS expression was determined using a modified version of a previously described method [45]. RAW 264.7 cells were seeded in 6-well plates at a density of 2 × 10^5^ cells per well and incubated overnight at 37°C with 5% CO_2_. Cells were treated with the BS solution at various concentrations and 0.5 μg/mL LPS for 4 h under the same conditions. After treatment, total RNA was extracted using NucleoZOL lysis buffer (Macherey-Nagel) according to the manufacturer’s protocol. The isolated RNA was quantified, and cDNA was synthesized using HiSenScript™ RH(-) RT PreMix Kit (Intron). Quantitative real-time PCR samples were analyzed using the CFX Connect Real-Time PCR System (Bio-Rad, USA) in the Bio-Health Materials Core-Facility, Jeju National University. Relative gene expression levels were calculated from the quantified amplification data. The cycling conditions were as follows: 95°C for 15 min, followed by 50 cycles of 95°C for 20 s and 60°C for 40 s. The primer sequences used for iNOS analysis are listed in Table S1.

### Inhibition of Chemokine Expression

Chemokine expression was determined using a modified version of a previously described method [46]. HaCaT cells were seeded in 6-well plates at a density of 1.5 × 10^5^ cells per well and incubated for 6 h at 37°C with 5% CO_2_. The medium was then replaced with serum-free DMEM, and cells were incubated for 18 h to induce serum starvation. Cells were stimulated with a combination of tumor necrosis factor-α (TNF-α) and interferon-γ (IFN-γ) at a final total concentration of 10 ng/mL (1:1 ratio, 5 ng/mL each), and simultaneously treated with the BS solution at non-cytotoxic concentrations. After 24 h, total RNA was extracted using NucleoZOL lysis buffer according to the manufacturer’s instructions. The extracted RNA was quantified, and cDNA was synthesized using the HiSenScript™ RH(-) RT PreMix Kit. Quantitative real-time PCR was performed using 2x Real-Time PCR Master Mix & Premix, and relative gene expression levels were calculated from the quantified amplification data. The cycling conditions were as follows: 95°C for 15 min, followed by 50 cycles of 95°C for 20 s and 60°C for 40 s. Silymarin (10 ppm) was used as a positive control. The primer sequences used for chemokine analysis are listed in Table S2.

### Statistical Analysis

All data are presented as mean ± SD from three independent measurements unless otherwise indicated. Statistical significance was determined using Student’s t-test by comparing each treatment group with the corresponding stimulus-treated control group. Differences were considered statistically significant at *p* < 0.05.

## Results

### Isolation and Screening of a Biosurfactant-Producing Lactic Acid Bacterium

After three consecutive enrichment cultures in customized MRS medium supplemented with rapeseed oil, the final enrichment culture was serially diluted and spread onto cMRS agar plates. Six LAB isolate candidates were selected as preliminary screening candidates based on visually distinct colony morphology and size and designated as J2K-1221, J2K-1223, J2K-1225, J2K-1227, J2K-1229, and J2K-1231. Each isolate was cultivated in cMRS broth, and surface activity was screened using an oil-spreading assay with two sample types prepared from each culture: (i) the oil-free cell-free supernatant (to evaluate extracellular activity) and (ii) PBS extracts from washed cells (to evaluate PBS-extractable, cell-associated activity). Among the six isolates, only the cell-free supernatant from strain J2K-1229 produced a clear oil-displacement zone indicative of strong surface activity (SI video), whereas none of the PBS buffer extracts exhibited detectable activity under the same assay conditions. The remaining five isolates (J2K-1221, J2K-1223, J2K-1225, J2K-1227, and J2K-1231) showed no detectable oil-spreading activity in either the cell-free supernatant or the PBS cell extract and were therefore not subjected to further taxonomic, genomic, or chemical characterization. Based on this screening, strain J2K-1229 was selected for subsequent identification, purification, and bioactivity evaluation.

### Phylogenetic Identification of Strain J2K-1229

**Fig. 1:**
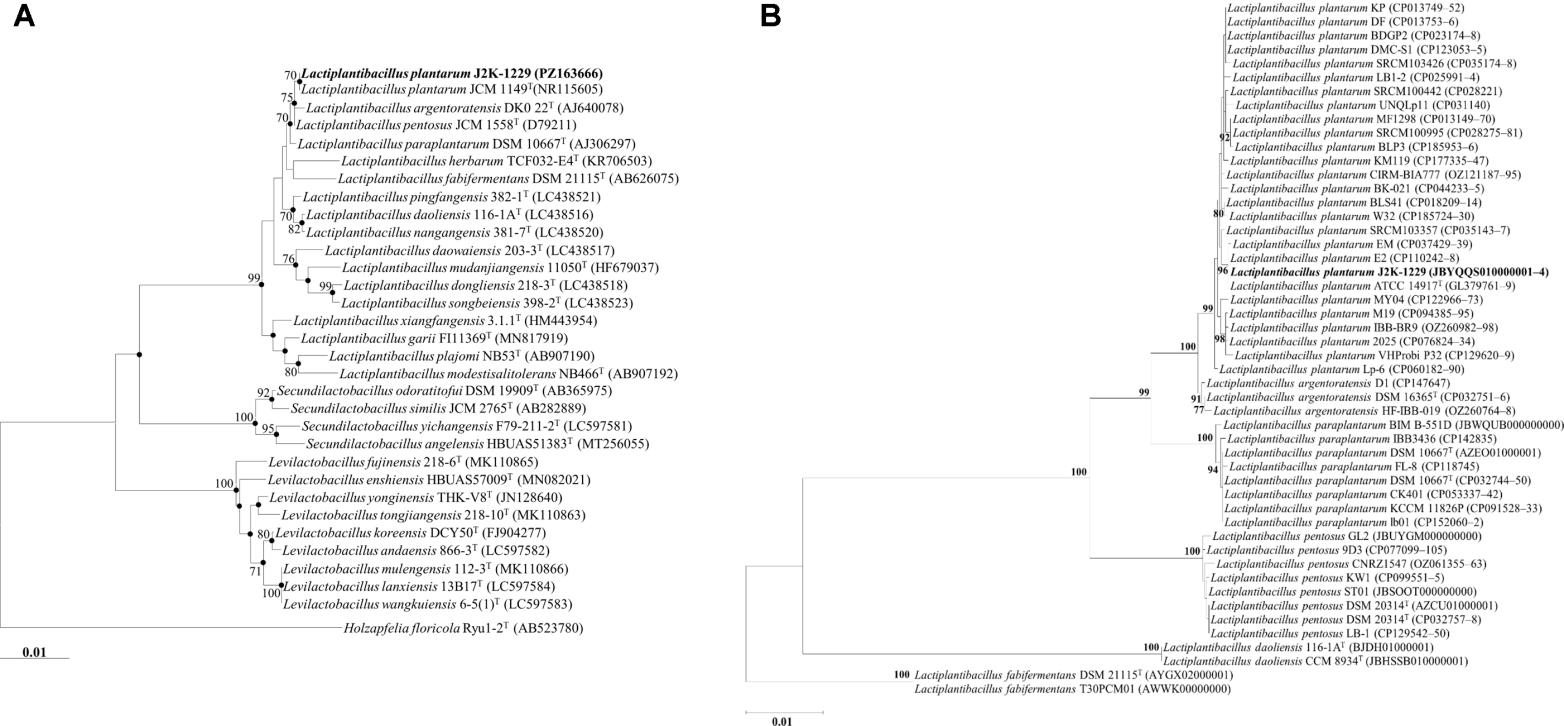
Phylogenetic placement of strain J2K-1229. (**A**) NJ tree based on 16S rRNA gene sequences showing the phylogenetic relationships of strain J2K-1229 and related taxa. Bootstrap values are shown on nodes (1000 replicates) when >70%. Filled circles indicate nodes also recovered by ML and MP analyses. *Holzapfelia floricola* Ryu1-2^T^ (AB523780) was used as an outgroup. Bar, 0.01 substitutions per nucleotide position. (**B**) Genome-based phylogenomic tree of strain J2K-1229 and closely related *Lactiplantibacillus* species inferred from a concatenated alignment of 120 single-copy bacterial marker genes (bac120; GTDB-Tk). Bootstrap values >70% (1,000 replicates) are indicated at nodes. Bar, 0.01 substitutions per amino acid position.

The 16S rRNA gene sequence of strain J2K-1229 was determined and compared with reference sequences. Comparative analysis showed that strain J2K-1229 was most closely related to *L. plantarum* JCM 1149^T^, sharing 100% sequence similarity. In the NJ phylogenetic tree constructed using aligned 16S rRNA gene sequences, strain J2K-1229 formed a lineage within the genus *Lactiplantibacillus* and clustered most closely with *L. plantarum* JCM 1149^T^ (Fig. 1A). This placement was consistently supported by phylogenetic analyses using MP and ML algorithms (Figs. S1 and S2), indicating that the isolate is *L. plantarum*.

### Whole-Genome Sequencing and Phylogenomic Identification of Strain J2K-1229

The de novo assembly yielded four circular replicons, consisting of one complete circular chromosome of 3,220 kb and three plasmid-like circular contigs of 70,732 bp, 38,916 bp, and 18,735 bp (Fig. S4). The total genome size was 3,348 kb, with a GC content of 44.3%. Genome quality assessment indicated high completeness, with 100.0% completeness and 0.52% contamination, satisfying the criteria for a high-quality genome (≥90% completeness and ≤10% contamination) [26]. Genome characteristics and functional genome-mining results are provided in Tables S3 and S4. AMRFinderPlus analysis detected no acquired antibiotic resistance and virulence-associated genes in the J2K-1229 genome.

The genome-based phylogenomic tree showed that strain J2K-1229 clustered with representative *L. plantarum* genomes, clearly separating from closely related *Lactiplantibacillus* species (Fig. 1B). The ANI and dDDH values between strain J2K-1229 and *L. plantarum* ATCC 14917^T^ were 99.6 and 98.4%, respectively, which were higher than the commonly used species delineation thresholds of 95–96% ANI and 70% dDDH [47]. These results supported the assignment of strain J2K-1229 to *L. plantarum*.

### Phenotypic Characterization of Strain J2K-1229

Phenotypic profiling using API ZYM and API 50 CHL was performed as supplementary characterization of strain J2K-1229. Because genome-based phylogeny, ANI, and dDDH provided the primary taxonomic evidence, the detailed API ZYM and API 50 CHL profiles were moved to Tables S5 and S6. Briefly, the API profiles were consistent with typical metabolic features of *Lactiplantibacillus* spp. and provided complementary support for the identification of strain J2K-1229 as *L. plantarum*.

### Emulsification Activity of the Culture Supernatant from Strain J2K-1229

The emulsification index was used to evaluate the emulsifying capacity of the biosurfactant-containing culture supernatant from strain J2K-1229 against a panel of oils relevant to cosmetic and personal care applications (Table 1). The cell-free supernatant formed measurable emulsions with all tested oils, with E_24_ values ranging from 26.67 ± 2.31% (moringa oil) to 41.33 ± 2.31% (avocado oil). Relatively high emulsification was observed with avocado oil (41.33 ± 2.31%) and argan oil (40.00 ± 4.00%). The supernatant also produced stable emulsions (> 32%) with jojoba, olive, soybean, meadowfoam seed, and rapeseed oils (Table 1). Lower E_24_ values (< 29%) were recorded with macadamia and evening primrose oils, as well as with moringa oil. As a benchmark control, 0.1% (w/v) Tween 80 generally showed higher E_24_ values across most oils (Table 1), confirming that the assay conditions could distinguish emulsification performance between the biological sample and a synthetic surfactant control.

### Chemical Characterization of the Purified Biosurfactant Fraction

**Fig. 2:**
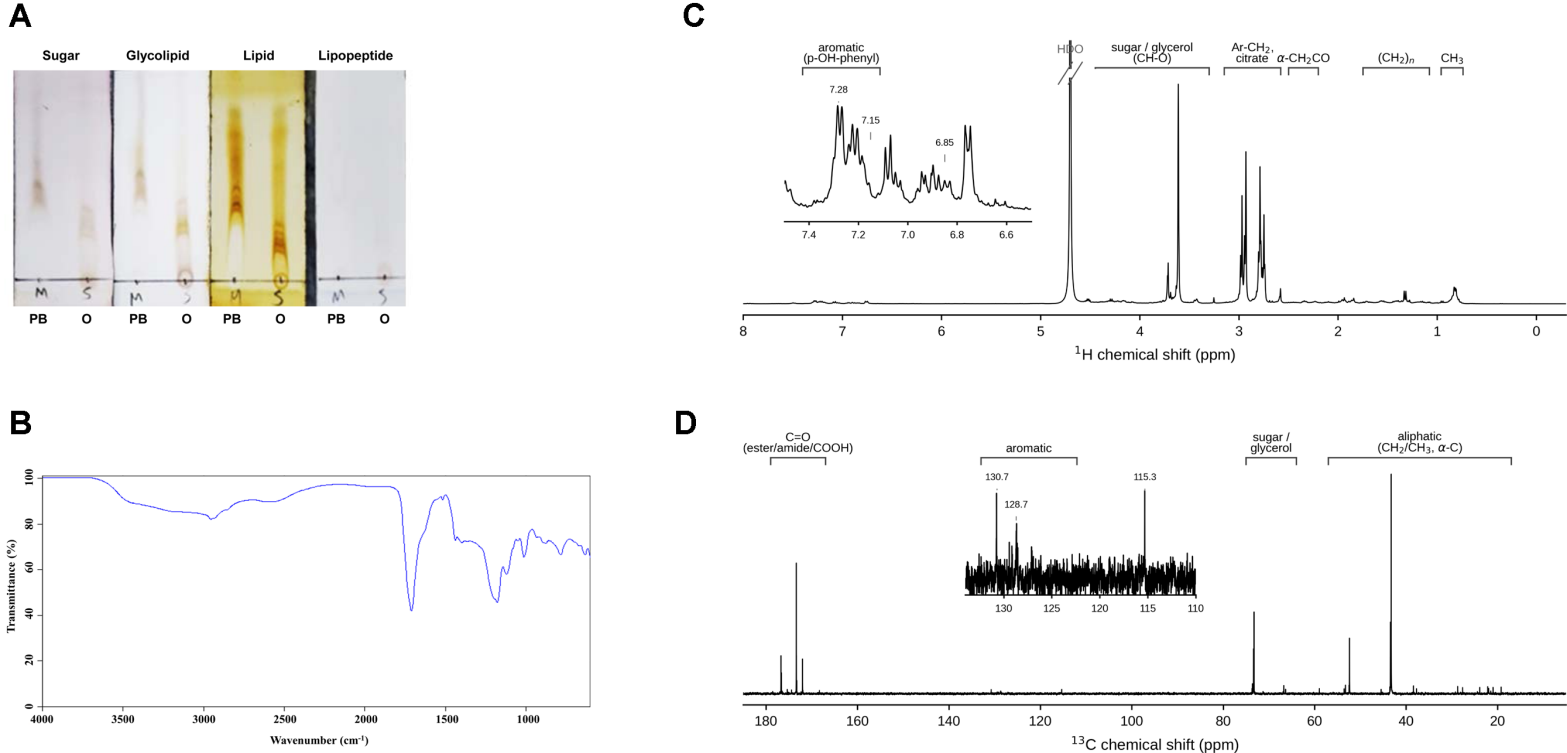
Chromatographic and spectroscopic characterization of the purified biosurfactant fraction produced by *Lactiplantibacillus plantarum* J2K-1229. (**A**) Thin-layer chromatography (TLC) profiles of the purified biosurfactant fraction (PB) visualized using anisaldehyde–sulfuric acid–glacial acetic acid, sulfuric acid in ethanol, iodine vapor, and ninhydrin reagents. O, other fractions obtained during normal-phase purification. (**B**) Fouriertransform infrared (FT–IR) spectrum of PB, showing bands for hydroxyl (–OH), aliphatic C–H, carbonyl (C=O; carboxylic acid and ester), and C–O/C–O–C groups; the assignments are summarized in Table 2. (**C**) One-dimensional ^1^H NMR spectrum of PB (with an expansion of the aromatic region), showing aliphatic ((CH_2_)_n_, CH_3_), α-CH_2_CO, glycerol/sugar (CH–O), and aromatic (para-hydroxyphenyl) resonances; HDO denotes the residual water signal. (**D**) One-dimensional ^13^C NMR spectrum of PB, showing carbonyl (ester/carboxylic acid), aromatic, glycerol/sugar, and aliphatic carbons. The aromatic signals in C and D are consistent with co-extracted phenyllactate-type metabolites.

Crude biosurfactants extracted from *L. plantarum* J2K-1229 cultures were purified by normal-phase chromatography, and the fraction retaining surface activity in the oil-spreading assay was collected as the purified biosurfactant preparation (PB). TLC analysis indicated that PB contained both carbohydrate- and lipid-associated components (Fig. 2A). The lipid-reactive stain gave the strongest signal, and the carbohydrate- and lipid-positive lanes resolved into multiple migrating bands, indicating that PB is a mixture of co-migrating species rather than a single purified compound (Fig. 2A).

FT–IR spectroscopy of PB showed bands consistent with hydroxylated, ester- and carboxylic acid-containing lipid molecules, including a broad –OH band at 3490 cm^−1^, aliphatic C–H stretching bands at 2954–2862 cm^−1^, a carbonyl (C=O) band at 1712 cm^−1^ consistent with overlapping carboxylic acid and ester carbonyls, methyl-related C–H bending at 1438–1380 cm^−1^, and a C–O/C–O–C band at 1180 cm^−1^ (Fig. 2B, Table 2). GC–FAME analysis revealed that the fatty acid fraction of PB was dominated by oleic acid (18:1 ω9c, 34.44%) and palmitic acid (16:0, 12.02%), with stearic acid (18:0, 5.03%) and several additional fatty acids present at lower abundances (Table 3). One-dimensional ^1^H and ^13^C NMR spectra of PB (Fig. 2C and 2D) were consistent with a complex, lipid-dominant mixture. The ^1^H spectrum was dominated by fatty-acyl resonances—a terminal methyl signal near 0.9 ppm, a bulk methylene envelope near 1.3 ppm, and allylic/α-carbonyl methylene signals near 2.0–2.3 ppm—together with glycerol/sugar oxymethine and oxymethylene signals between ~3.3 and ~4.3 ppm; correspondingly, the ^13^C spectrum showed aliphatic carbons at ~14–34 ppm, glycerol/sugar carbons at ~60–75 ppm, and multiple carbonyl resonances at ~172–178 ppm consistent with overlapping ester and carboxylic acid carbonyls. In addition, both spectra contained signals diagnostic of a para-substituted phenolic ring (^1^H ~6.85, 7.15, and 7.28 ppm; ^13^C ~115, 128, and 130 ppm), indicating co-extracted aromatic constituents consistent with phenyllactic acid/4-hydroxyphenyllactic acid-type metabolites rather than a glycolipid headgroup [14].

Overall, the combined TLC, FT–IR, GC–FAME, HPAEC–PAD, and NMR data indicate that PB is a structurally complex, lipid-dominant biosurfactant preparation rather than a single pure compound. It comprises a glucose-based glycolipid/glycerolipid component together with free fatty acids and co-extracted low-molecular-weight aromatic metabolites (phenyllactate-type), and the single monosaccharide (fructose) detected by HPAEC–PAD is interpreted with caution—most plausibly as co-extracted free fructose rather than a glycosidically bound headgroup, as addressed by the genome-based analysis below.

### Genomic Features and Functional Genome Mining

**Fig. 3:**
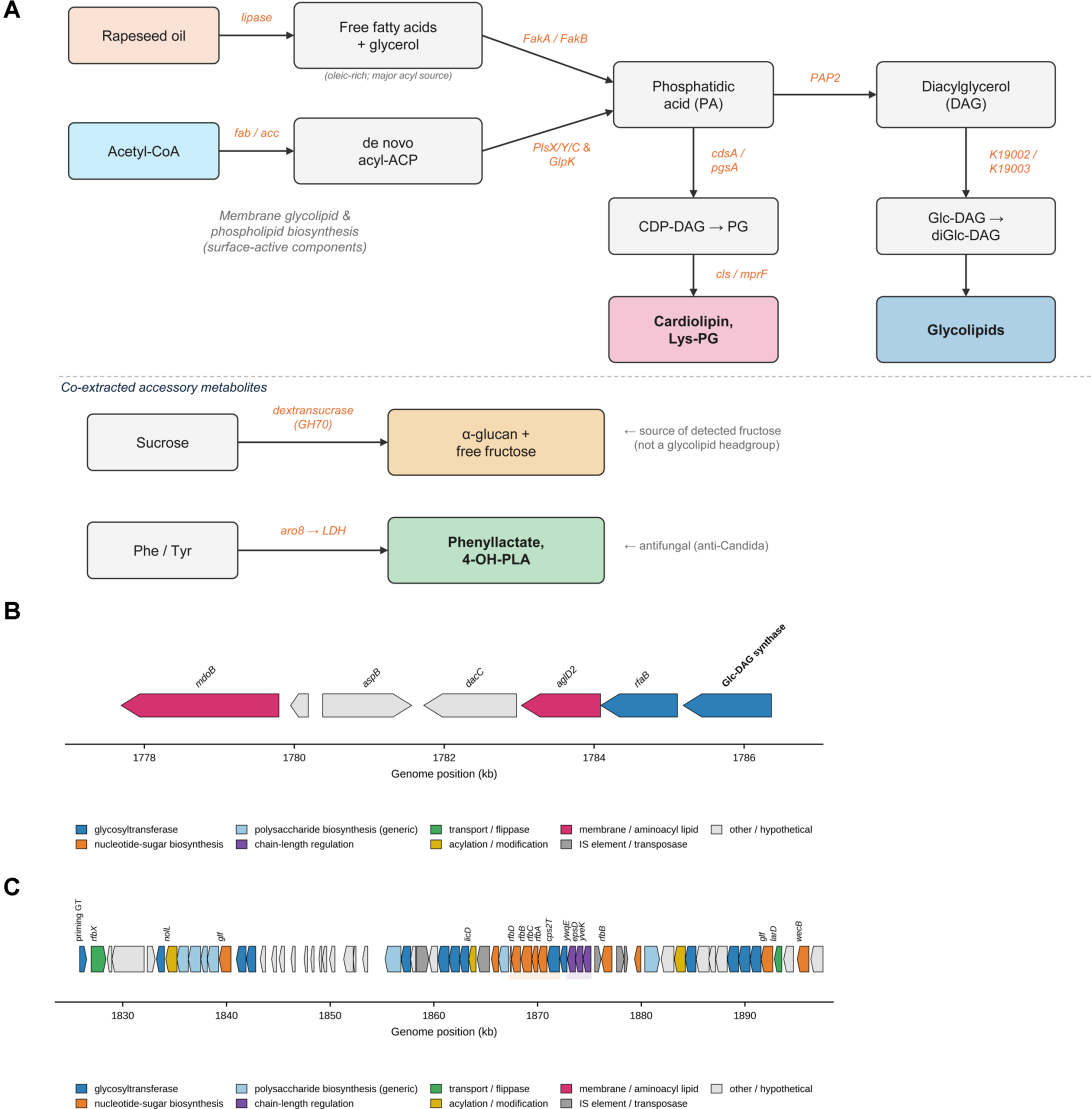
Genome-supported biosynthesis model and associated gene clusters in *Lactiplantibacillus plantarum* J2K-1229. (**A**) Proposed model for membrane-lipid and glucose-based glycolipid biosynthesis together with co-extracted accessory metabolites. Boxes denote metabolites or intermediates, and colored labels denote the corresponding enzymes/genes (KEGG/Bakta annotations); the lower section shows free fructose released from sucrose by a GH70 glucansucrase (dextransucrase)—the most likely source of the detected monosaccharide—and phenyllactate/4-hydroxyphenyllactate derived from aromatic amino acids. (**B**) Gene-arrow map of the glucosyl-diacylglycerol (glycolipid) biosynthesis module (genome position ~1,778–1,786 kb), including the 1,2-diacylglycerol 3-glucosyltransferase (Glc-DAG synthase) and *rfaB*. (**C**) Gene-arrow map of the exopolysaccharide/capsular-polysaccharide biosynthesis cluster (~1,828–1,896 kb). In (**B**) and (**C**), arrows represent genes oriented in the direction of transcription and are colored by predicted functional category as shown in the key; maps were drawn from the Bakta annotation.

Functional genome mining identified several candidate genes spanning fatty-acyl supply, glycerolipid and membrane-lipid assembly, glycolipid-headgroup formation, and exopolysaccharide biosynthesis (Table S4), and a putative glycolipid-precursor and membrane-lipid biosynthesis model was reconstructed using KEGG Mapper (Fig. 3A). Genes associated with *de novo* fatty acid biosynthesis (AccABCD, FabD, FabH, FabF, FabG, FabZ, FabI, and AcpP) were identified, supporting the genomic capacity to generate fatty-acyl pools; in addition, the strain encoded a route for salvaging exogenous fatty acids from the rapeseed-oil substrate, comprising secreted lipase/esterase genes and a fatty-acid kinase system (*fakA* with DegV/FakB-family fatty-acid-binding proteins) that re-activates free fatty acids for membrane-lipid synthesis. In addition, glycerolipid-related genes, including *plsY* and *plsC*, were detected, together with a glycerol kinase (*glpK*) supplying glycerol-3-phosphate, suggesting the formation of LPA and PA, and the PA-to-DAG conversion step was supported by PAP2-family phosphatase candidates (*pgpB*- and *yodM*-like genes).

Consistent with the established glycolipid biochemistry of *L. plantarum* [12], the genome encoded glucosyltransferases predicted to assemble glucose-based glycolipids, namely a 1,2-diacylglycerol 3-glucosyltransferase (Glc-DAG synthase, K19002; J2K1229_01742), a diglucosyl-diacylglycerol synthase (J2K1229_01280), and an adjacent glycosyltransferase (*rfaB*); a phosphatidylglycerol lysyltransferase (*mprF/aglD2*) accounting for lysyl-phosphatidylglycerol membrane lipids was also present. Critically, no fructosyltransferase or fructolipid-synthase gene was identified, indicating that the membrane glycolipids of strain J2K-1229 are glucose-based rather than fructose-based. The single monosaccharide (fructose) detected by HPAEC–PAD is therefore most plausibly attributable to a glucansucrase (dextransucrase; GH70 family, J2K1229_01942), which liberates free fructose as a by-product of extracellular glucan synthesis from sucrose; such free fructose is readily co-purified and reconciles the glucose-based genomic potential with the fructose detected biochemically. Finally, a large (~70 kb) exopolysaccharide/capsular-polysaccharide biosynthesis cluster—comprising a priming glycosyltransferase, multiple chain-extending glycosyltransferases, a dTDP-L-rhamnose pathway, and Wzc/Wzb-type chain-length regulation—was identified, typical of lactic acid bacteria [13] and consistent with the emulsifying, bioemulsifier-like behavior of the culture supernatant (Fig. 3B and 3C).

### Antimicrobial and Anti-inflammatory Activities of the Biosurfactant Preparation

The bioactivities of the biosurfactant preparation were evaluated using antimicrobial assays and cell-based inflammatory models. Antimicrobial activity was assessed by disk diffusion using *S. aureus* ATCC 6538, *E. coli* ATCC 8739, *P. aeruginosa* ATCC 9027, and *Candida albicans* ATCC 10231 as test organisms. Purified biosurfactant fraction was dissolved in sterile PBS and tested at 1%, 2%, 5%, and 10% (w/v), with PBS used as a negative control. The biosurfactant preparation displayed pronounced antifungal activity against *C. albicans* ATCC 10231 (Fig. S5). Clear inhibition zones were observed at 2%, 5%, and 10% (w/v), whereas a smaller inhibition zone was observed at 1% (w/v). In contrast, antibacterial activity against *S. aureus*, *E. coli*, and *P. aeruginosa* was comparatively weak and was only evident at higher biosurfactant concentrations (5% and 10% (w/v)) in this assay format (Fig. S5).

For cell-based assays, a biosurfactant solution (BS solution) was prepared by dissolving purified biosurfactant fraction in distilled water and diluted to working concentrations. Cell viability was examined by MTT assay in RAW 264.7 macrophages and HaCaT keratinocytes following 24 h exposure to BS solution at 5, 10, 25, 50, 100, and 200 ppm. Under these conditions, the BS solution showed no detectable cytotoxicity in either cell line across the tested concentration range (Fig. S6), supporting the use of these concentrations for inflammatory response assays.

**Fig. 4:**
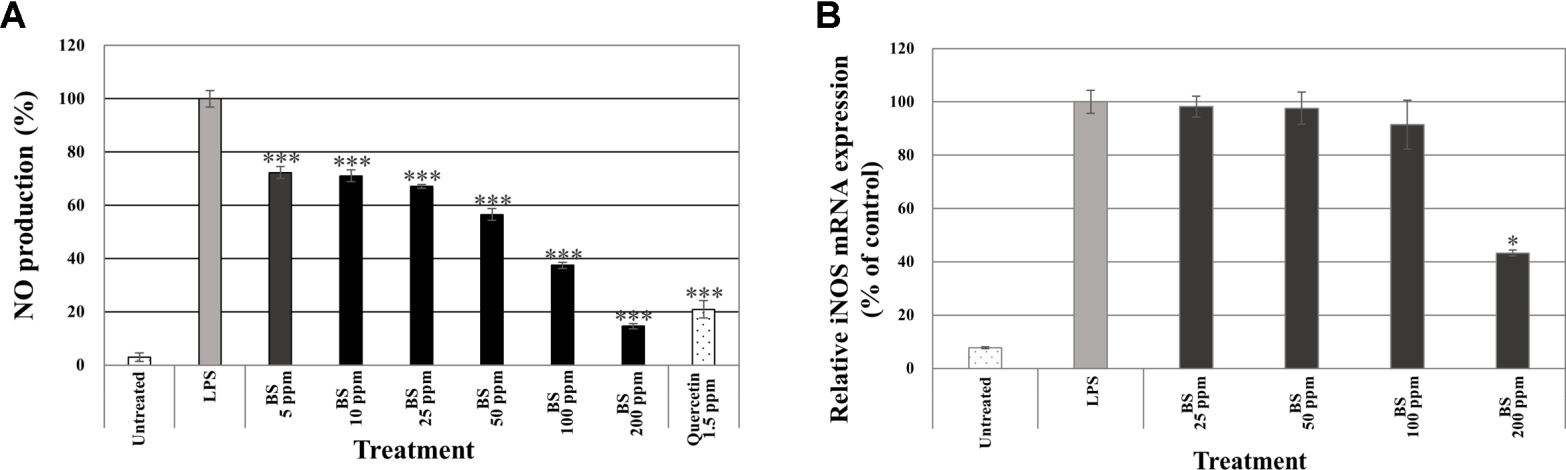
Inhibitory effects of the biosurfactant solution on inflammatory mediators in LPS-stimulated RAW 264.7 macrophages. (**A**) Nitric oxide (NO) production in RAW 264.7 cells treated with different concentrations of the biosurfactant solution (BS solution) and stimulated with lipopolysaccharide (LPS). Quercetin (1.5 ppm) was used as a positive control. (**B**) Relative mRNA expression of inducible nitric oxide synthase (iNOS) in RAW 264.7 cells treated with BS solution and stimulated with LPS. Gene expression levels were normalized to the control and expressed as a percentage of the control. RAW 264.7 cells were pretreated with BS solution at the indicated concentrations and subsequently stimulated with LPS. Values are presented as mean ± SD (n = 3). Statistical significance was determined using Student’s t-test relative to the LPS-treated control (*p < 0.05, **p < 0.01, ***p < 0.001).

In LPS-stimulated RAW 264.7 macrophages, BS solution reduced NO production in a concentration-dependent manner (Fig. 4A). The strongest inhibition of NO production was observed at 200 ppm, reaching an inhibition rate of 88.07% relative to the LPS-stimulated control. Consistently, BS solution reduced iNOS mRNA expression in a concentration-dependent manner (Fig. 4B), with an inhibition rate of 61.47% at 200 ppm.

**Fig. 5:**
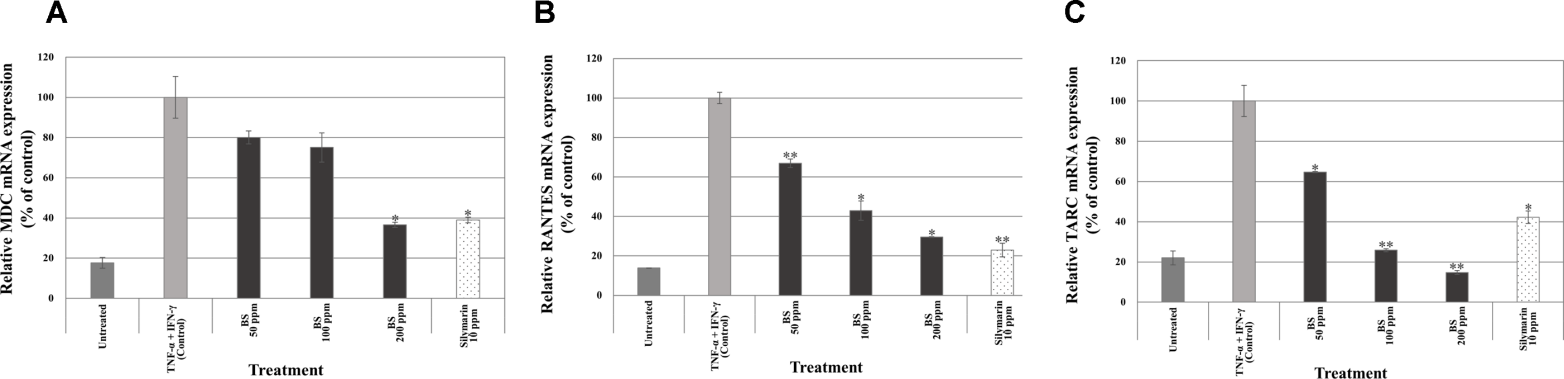
Effects of the biosurfactant preparation on chemokine expression in cytokine-stimulated HaCaT keratinocytes. (**A**) Relative mRNA expression levels of macrophage-derived chemokine (MDC). (**B**) Relative regulated on activation, normal T cell expressed and secreted (RANTES) mRNA expression. (**C**) Relative thymus and activation-regulated chemokine (TARC) mRNA expression. HaCaT cells were treated with the biosurfactant solution (BS solution) at 50, 100, and 200 ppm and stimulated with tumor necrosis factor-α (TNF-α) and interferon-γ (IFN-γ); silymarin (10 ppm) served as a positive control. Expression was quantified by quantitative real-time PCR and normalized to the cytokine-stimulated control. Values are mean ± SD (n = 3); statistical significance was determined using Student’s t-test versus the cytokine-stimulated control (*p < 0.05, **p < 0.01, ***p < 0.001).

In TNF-α and IFN-γ-stimulated HaCaT keratinocytes, BS solution decreased the mRNA expression of the chemokines MDC, RANTES, and TARC in a concentration-dependent manner (Fig. 5). At 200 ppm, the relative mRNA expression levels were reduced to 36.58% (MDC), 29.51% (RANTES), and 14.72% (TARC) compared with the stimulated control (Fig. 5). Overall, within a non-cytotoxic concentration range, the biosurfactant preparation from *L. plantarum* J2K-1229 showed strong antifungal activity against *C. albicans* in disk diffusion assays and suppressed inflammatory mediator outputs in macrophage- and keratinocyte-based *in vitro* models (Figs. 4, 5, and S5).

## Discussion

This study identified an olive-derived *L. plantarum* strain J2K-1229 that produced extracellular material with surface/interfacial activity under rapeseed-oil-containing culture conditions. This activity was detected in the cell-free supernatant, whereas PBS extracts from cells showed no detectable activity, indicating that the surface-active material was primarily extracellular under the conditions tested. The cell-free supernatant emulsified multiple cosmetic-relevant oils, and the chromatographically enriched active preparation showed anti-*Candida* and anti-inflammatory activities in vitro. These findings suggest that strain J2K-1229 produces a multifunctional biosurfactant preparation with potential relevance to food- and cosmetic-related applications. However, the E24 assay primarily reflects emulsion formation and stability and does not directly quantify classical surfactant parameters such as surface tension reduction or critical micelle concentration (CMC). Therefore, the preparation should be interpreted as an extracellular surface/interfacial-active material with possible bioemulsifier-type contribution, rather than as a fully defined low-molecular-weight biosurfactant.

The chemical data indicate that the active fraction is best described as a structurally complex, lipid-dominant biosurfactant preparation rather than a single purified glycolipid molecule. TLC showed carbohydrate- and lipid-positive signals and was ninhydrin-negative, but the presence of multiple bands indicated that the preparation was not homogeneous. FT–IR and GC–FAME supported the presence of lipid-associated constituents, with oleic acid and palmitic acid as major fatty acids. HPAEC–PAD detected fructose as the only monosaccharide in the hydrolysate, but this result alone does not establish fructose as the glycosidically bound headgroup of the principal glycolipid. One-dimensional ^1^H and ^13^C NMR confirmed abundant fatty-acyl and glycerol/sugar resonances but additionally revealed aromatic signals consistent with co-extracted phenyllactic acid/4-hydroxyphenyllactic acid-type metabolites. Therefore, the active preparation likely contains glycolipid/glycerolipid-related components, fatty acids, fructose or fructose-derived material, and low-molecular-weight aromatic metabolites. This compositional complexity is important because the observed bioactivities cannot yet be attributed to a single glycolipid component.

Genome-based functional analysis reconciled these chemical observations with the strain’s biosynthetic potential. The genome encoded genes associated with fatty-acyl supply, glycerolipid and membrane-lipid assembly, glucose-based glycolipid formation, exopolysaccharide/capsular-polysaccharide biosynthesis, and phenyllactic acid-related metabolism. In particular, the presence of glucosyl-diacylglycerol-related glycosyltransferases was consistent with the known capacity of *L. plantarum* to produce glucose-based membrane glycolipids [12]. In contrast, no clear fructosyltransferase or fructolipid-synthase gene was identified. Therefore, the fructose detected by HPAEC–PAD is more cautiously interpreted as co-extracted free fructose, possibly released during glucansucrase-mediated sucrose metabolism, rather than as direct evidence of a fructosylated glycolipid headgroup [48]. The GC–FAME profile, dominated by oleic acid, also suggests that fatty acids derived from the rapeseed-oil substrate may contribute to the acyl composition of the preparation. In addition, the large exopolysaccharide/capsular-polysaccharide biosynthesis cluster may help explain the emulsifying, bioemulsifier-like behavior of the culture supernatant. Thus, the reconstructed pathway should be regarded as a genome-supported biosynthetic interpretation of the preparation, not as definitive structural proof of a specific glycolipid molecule.

Functionally, the biosurfactant preparation showed a clear antifungal preference, with pronounced inhibition of *C. albicans* but comparatively weak antibacterial activity. This selectivity is consistent with the presence of co-extracted phenyllactic acid-type metabolites, which are known antifungal compounds produced by *L. plantarum* [14, 15]. Accordingly, the anti-*Candida* activity cannot be assigned specifically to the glycolipid component. Instead, the observed activity may result from the combined effects of phenyllactate-type metabolites, fatty acid/lipid components, and surface-active constituents in the multicomponent preparation. This represents an important limitation of the present study and highlights the need for component-resolved antimicrobial testing.

The preparation also reduced NO production and iNOS expression in LPS-stimulated RAW 264.7 macrophages and suppressed MDC/CCL22, RANTES/CCL5, and TARC/CCL17 expression in TNF-α/IFN-γ-stimulated HaCaT keratinocytes without detectable cytotoxicity up to 200 ppm. These results indicate anti-inflammatory potential in macrophage- and keratinocyte-based *in vitro* models. However, because the preparation is multicomponent and the present assays mainly measured endpoint responses at the mediator or mRNA level, the active constituents and underlying signaling mechanisms remain unresolved. Protein-level validation and component-resolved testing would be needed to determine whether the observed effects are driven by glycolipid-related constituents, phenyllactate-type metabolites, fatty acids, or their combined action.

Overall, this study demonstrates that olive-derived *L. plantarum* J2K-1229 produces an extracellular, multicomponent, glycolipid-containing biosurfactant preparation with emulsifying, anti-*Candida*, and anti-inflammatory activities. The main limitation is that the active preparation was not resolved to individual molecules; therefore, the chemical identity of the principal surface-active component and the compounds responsible for the observed bioactivities remain to be determined. Future work should focus on intact-molecule liquid chromatography–tandem mass spectrometry (LC–MS/MS), 2D NMR, quantitative surfactant benchmarks such as surface/interfacial tension and CMC, and bioassay-guided fractionation to define the active constituents and evaluate formulation applicability.

## Conclusion

In this study, an olive-derived LAB strain, *L. plantarum* J2K-1229, was shown to produce extracellular material with surface/interfacial activity when cultivated in a rapeseed oil-containing medium. The purified biosurfactant fraction was characterized as a structurally complex, lipid-dominant biosurfactant preparation by complementary chromatographic and spectroscopic analyses, with oleic and palmitic acids as the major fatty acids and NMR signals consistent with co-extracted phenyllactic acid-type metabolites. Genome mining indicated a glucose-based glycolipid biosynthetic capacity with no fructosyltransferase, supporting the interpretation that the fructose detected by HPAEC–PAD may represent co-extracted free fructose rather than a fructosylated glycolipid headgroup.

Functionally, the biosurfactant preparation exhibited broad emulsification activity across cosmetic-relevant plant oils and pronounced antifungal activity against *Candida albicans*. In addition, the BS solution showed anti-inflammatory responses in macrophage and keratinocyte *in vitro* models without detectable cytotoxicity up to 200 ppm. Taken together, the combined interfacial activity and bioactivities suggest that this preparation could be developed as a multifunctional bio-based ingredient for cosmetic and personal-care formulations, potentially serving as an emulsifying/co-surfactant component with yeast-control and skin-soothing supportive functions. However, because the active preparation was not resolved to individual molecules, future studies should focus on intact-molecule LC–MS/MS, 2D NMR, quantitative surfactant benchmarks, and bioassay-guided fractionation to define the active constituents and evaluate formulation applicability.

## Supplemental Materials

Supplementary data for this paper are available on-line only at http://jmb.or.kr.



## Figures and Tables

**Table 1:** Emulsification index after 24 h (E24) of different oils using the cell-free supernatant of *Lactiplantibacillus plantarum* J2K-1229.

Type of Oils	Cell-free Supernatant (J2K-1229)	0.1% Tween 80^a^
Jojoba	36 ± 4.00	62.67 ± 2.31
Olive	38.67 ± 2.31	61.33 ± 2.31
Avocado	41.33 ± 2.31	58.67 ± 2.31
Argan	40 ± 4.00	57.33 ± 2.31
Soybean	38.67 ± 2.31	58.67 ± 2.31
Macadamia	28 ± 4.00	30.67 ± 2.31
Evening primrose	29.33 ± 2.31	58.67 ± 2.31
Moringa	26.67 ± 2.31	57.33 ± 2.31
Meadowfoam seed	32 ± 4.00	58.67 ± 2.31
Rapeseed	33.33 ± 2.31	58.67 ± 2.31

The index was determined using the cell-free supernatant of strain J2K-1229 against various oils relevant to cosmetic and personal care application.

^a^Tween 80 (0.1%, w/v) was used as a synthetic surfactant control. Values are presented as mean ± SD (n = 3).

**Table 2:** Major FT−IR absorption bands and functional group assignments of the purified biosurfactant fraction produced by *Lactiplantibacillus plantarum* J2K-1229.

Wavelength (cm^−1^)	Functional group assignment
3490	O−H stretch (hydroxyl)
2954−2862	C−H stretch, aliphatic (CH_3_, CH_2_)
1712	C=O stretch (carboxylic acid and ester carbonyl)
1438−1380	C−H bending (CH_2_/CH_3_)
1180	C−O / C-O-C stretch (ester and sugar)

**Table 3:** Fatty acid composition of the purified biosurfactant fraction produced by *Lactiplantibacillus plantarum* J2K-1229 as determined by GC−FAME analysis.

Fatty Acid	Composition (%)^a^
Oleic acid (18:1 ω9c)	34.44
Palmitic acid (16:0)	12.02
Stearic acid (18:0)	5.03
Vaccenic acid (18:1 ω7c)	4.72
17-Methyl-octadecanoic acid (19:0 iso)	4.50
Paullinic acid (20:1 ω7c)	4.44
γ-Linolenic acid (18:3 ω6c)	4.17
Others^b^	18.77

Values represent mean percentages from triplicate analyses.

^a^Fatty acid composition is expressed as the relative percentage of total detected fatty acids. ^b^Fatty acids present at levels below 3.0% individually are grouped as “Others.”
