# Supplementary material for: Compositional and Functional Characterization of Glycolipid Biosurfactants from an Olive-Derived Lactiplantibacillus plantarum
Source: J Microbiol Biotechnol. 2026 Aug 10;36:e2606027. doi: 10.4014/jmb.2606.06027 (PMC13456973; doi:10.4014/jmb.2606.06027)
Supplement: Supplementary file 1 [file jmb-36-e2606027-supple.pdf]

J. Microbiol. Biotechnol. 2026. 36: e2606027

https://doi.org/10.4014/jmb.2606.06027

Supplementary Information

Compositional and Functional Characterization of Glycolipid Biosurfactants from an Olive-Derived *Lactiplantibacillus plantarum*

Jong Woo Hyeon[1,2], Min Hui Jeon[1], Jun-Tae Bae[1], and Man-Young Jung[2,3,4,5*]

[1]R&D Center, J2KBIO, Cheongju 28104, Republic of Korea

[2]Interdisciplinary Graduate Program in Advanced Convergence Technology and Science, Jeju National University, Jeju 63243, Republic of Korea

[3]Department of Biology Education, Jeju National University, Jeju 63243, Republic of Korea

[4]Bio-Health Materials Core-Facility Center, Jeju National University, Jeju 63243, Republic of Korea

[5]Jeju Microbiome Center, Jeju National University, Jeju 63243, Republic of Korea

[*]Corresponding author: [myjung@jejunu.ac.kr]

Table S1. Primers used for iNOS expression analysis by qPCR in this study.

Primer	Sequence (5′-3′)	
	Forward	Reverse
GAPDH	CATCACTGCCACCCAGAAGACTG	ATGCCAGTGAGCTTCCCGTTCAG
iNOS	GAGACAGGGAAGTCTGAAGCAC	CCAGCAGTAGTTGCTCCTCTTC

Table S2. Primers used for chemokine expression analysis by qPCR in this study.

Primer	Sequence (5′-3′)	
	Forward	Reverse
GAPDH	GGAGCGAGATCCCTCCAAAAT	ATGCCAGTGAGCTTCCCGTTCAG
MDC	AGGACAGAGCATGGCTCGCCTACAGA	TAATGGCAGGGAGCTAGGGCTCCTGA
RANTES	CCCCGTGCCGAGATCAAGGAGTATTT	CGTCCAGCCTGGGGAAGGTTTTTGTA
TARC	ATGGCCCCACTGAAGATGCT	TGAACACCAACGGTGGAGGT

Table S3. Genome characteristics of *Lactiplantibacillus plantarum* J2K-1229 from Bakta annotation.

Genome features	Activity
Genome size (bp)	
Chromosome	3,220,291
Plasmid 1	70,732
Plasmid 2	38,916
Plasmid 3	18,735
Genome coverages	158×, 321×, 192×, 71×
GC content (%)	44.3
CDSs	3,145
tRNAs	71
rRNAs	16
ncRNAs	9
Pseudogenes	19

Table S5. API ZYM enzymatic profiles of strain J2K-1229.

Enzyme	Activity	Enzyme	Activity
Alkaline phosphatase	+	Naphtol-AS-BI-phosphohydrolase	+
Esterase (C4)	+	*α*-galactosidase	+
Esterase lipase (C8)	+	*β*-galactosidase	+
Lipase (C14)	+	*β*-glucuronidase	+
Leucine arylamidase	+	*α*-glucosidase	+
Valine arylamidase	+	*β*-glucosidase	+
Crystine arylamidase	+	*N*-acetyl-*β*-glucosaminidase	+
Trypsin	-	*α*-mannosidase	-
α-chymotrypsin	-	*α*-fucosidase	-
Acid phospatase	+		

Table S6. API 50 CHL carbohydrate fermentation profiles of strain J2K-1229.

Substrate	Fermentation	Substrate	Fermentation
Glycerol	-	Salicin	+
Erythritol	-	D-Cellobiose	+
D-Arabinose	-	D-Maltose	+
L-Arabinose	+	D-Lactose	+
D-Ribose	+	D-Melibiose	+
D-Xylose	-	D-Sucrose	+
L-Xylose	-	D-Trehalose	+
D-Adonitol	-	Inulin	+
Methyl-β-D-xylopyranoside	-	D-Melezitose	+
D-Galactose	+	D-Raffinose	+
D-Glucose	+	Starch	-
D-Fructose	+	Glycogen	-
D-Mannose	+	Xylitol	-
L-Sorbose	-	Gentiobiose	+
L-Rhamnose	+	D-Turanose	+
Dulcitol	-	D-Lyxose	-
Inositol	-	D-Tagatose	-
D-Mannitol	+	D-Fucose	-
D-Sorbitol	-	L-Fucose	-
Methyl-α-D-mannopyranoside	+	D-Arabitol	-
Methyl-α-D-glucopyranoside	-	L-Arabitol	-
N-acetyl-D-glucosamine	+	Potassium gluconate	+
Amygdalin	+	Potassium 2-ketogluconate	-
Arbutin	+	Potassium 5-ketogluconate	-
Esculin ferric citrate	+		

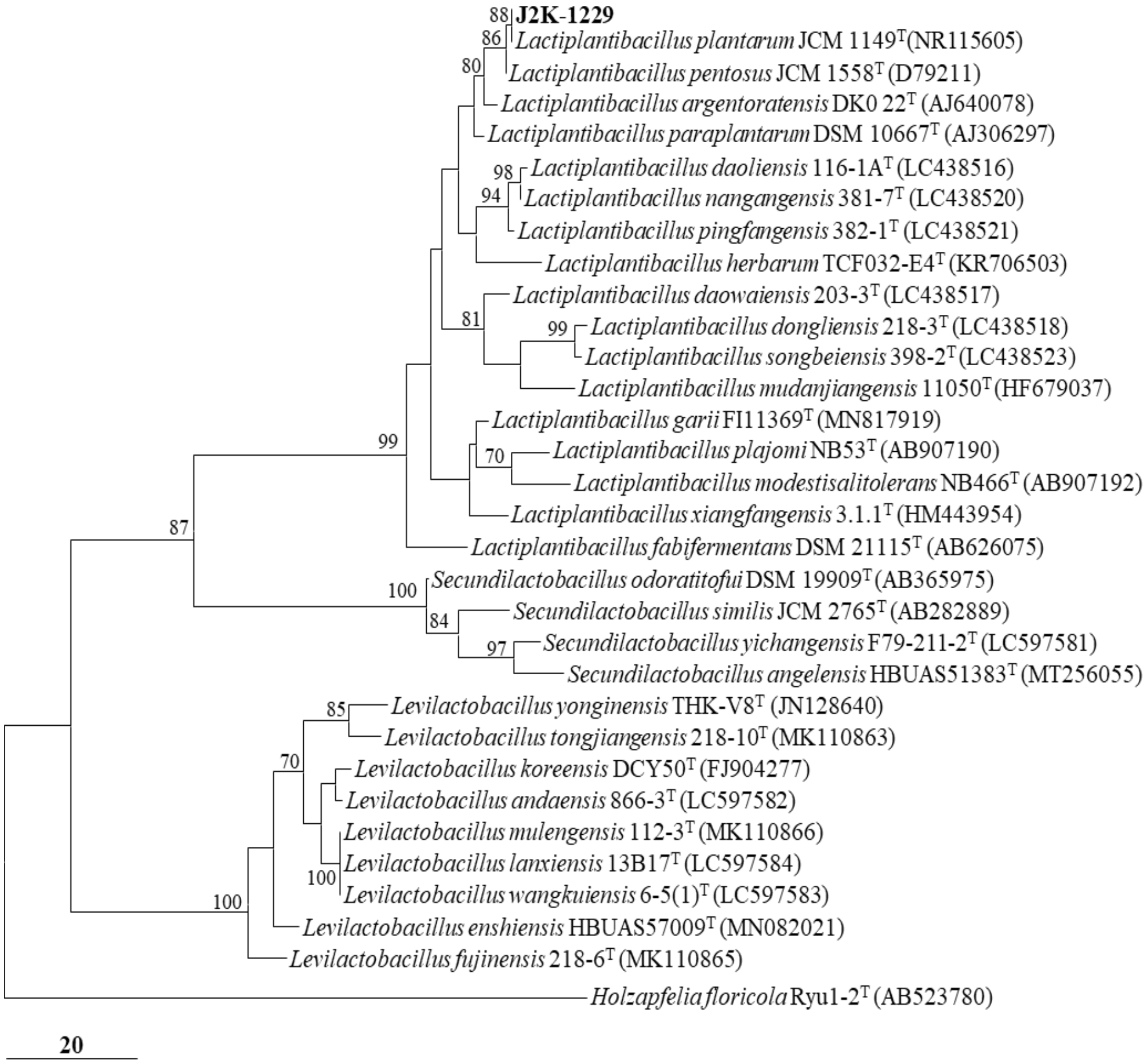

Fig. S1. MP tree based on 16S rRNA gene sequences showing the phylogenetic relationships of strain J2K-1229 and related taxa. Bootstrap values are shown on nodes in percentages of 1000 replicates, only when values were over 70%.

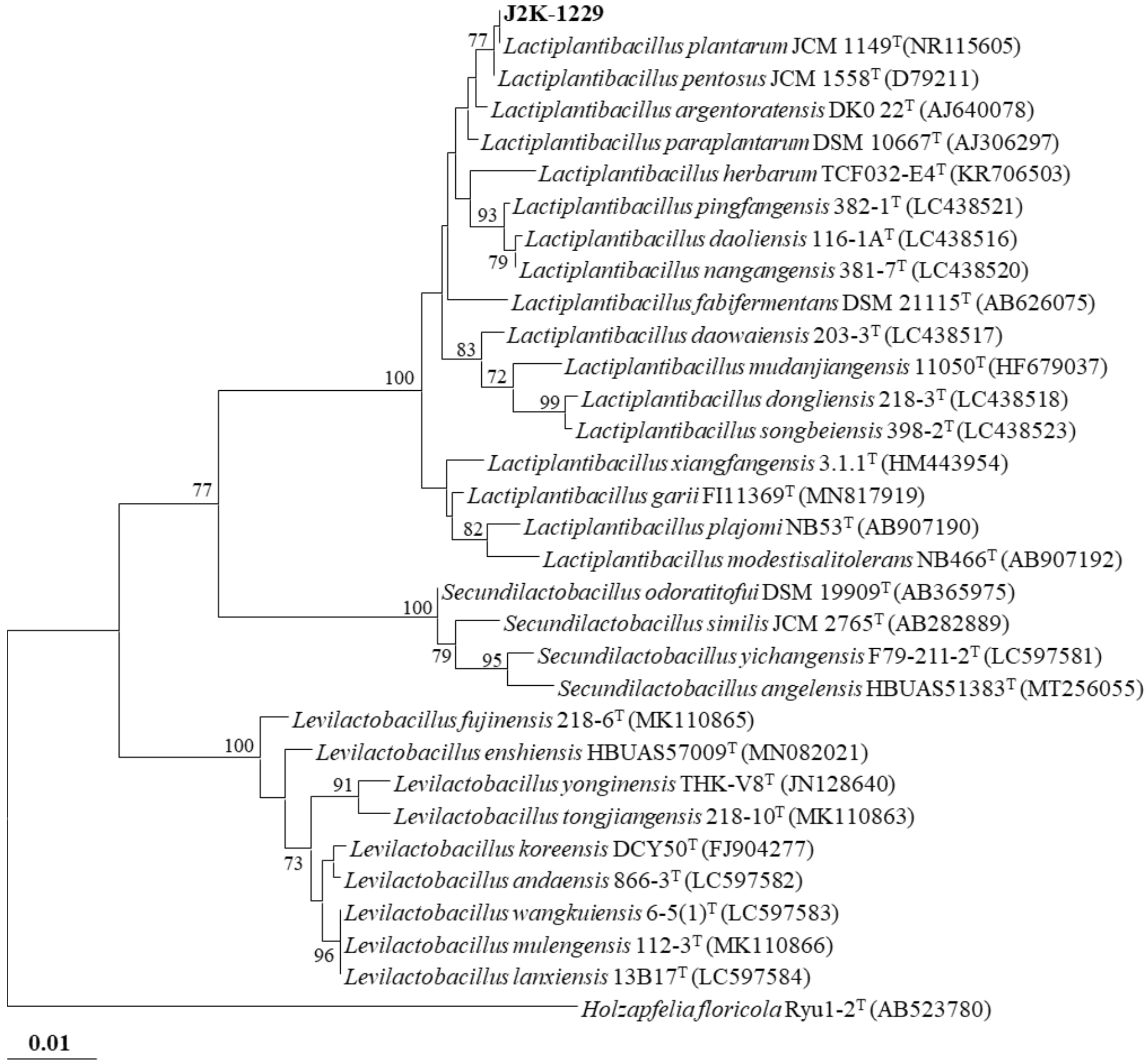

Fig. S2. ML tree based on 16S rRNA gene sequences showing the phylogenetic relationships of strain J2K-1229 and related taxa. Bootstrap values are shown on nodes as percentages based on 1000 replicates, only when values exceed 70%.

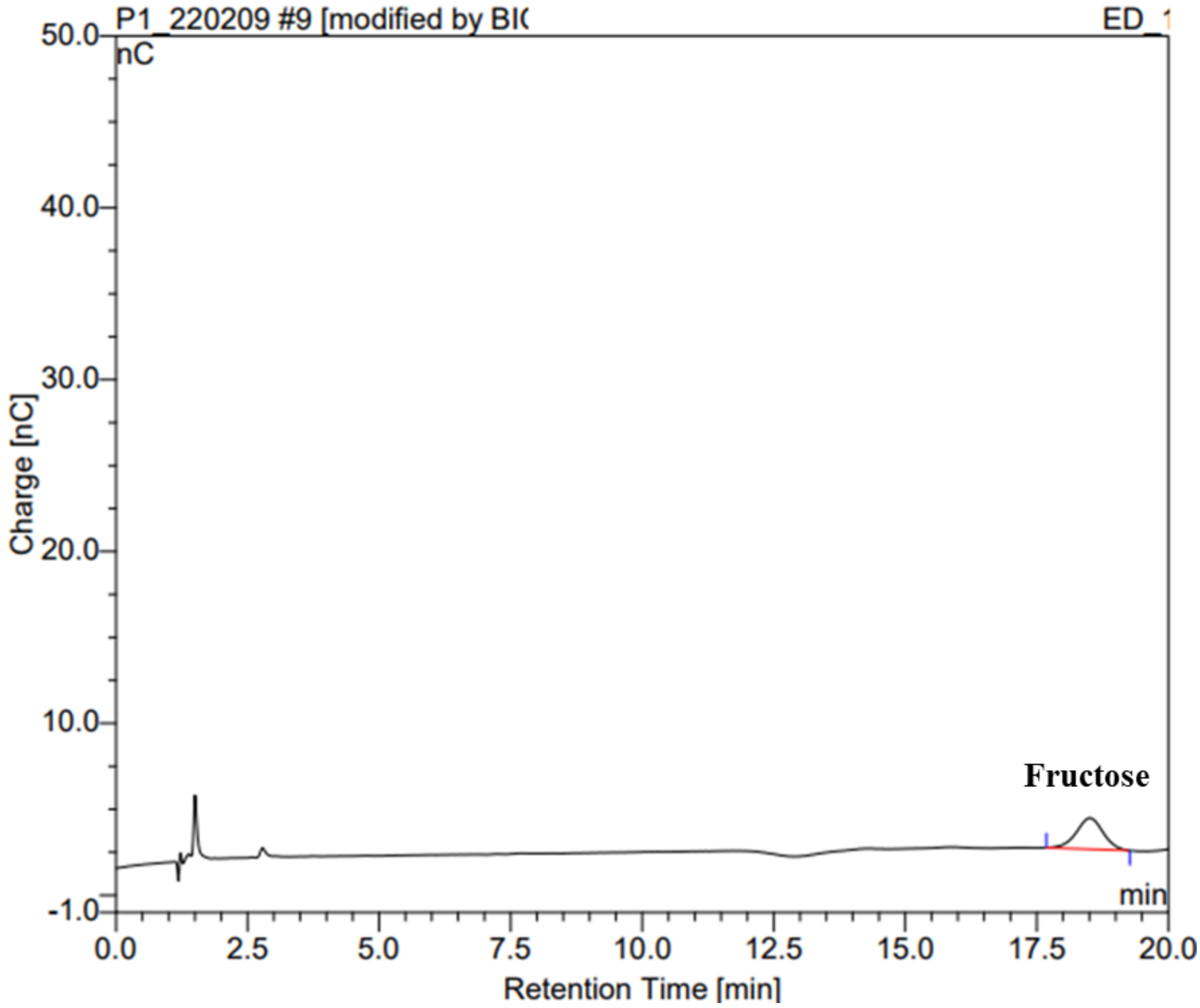

Fig. S3. HPAEC–PAD chromatogram of the purified biosurfactant fraction produced by strain J2K-1229.

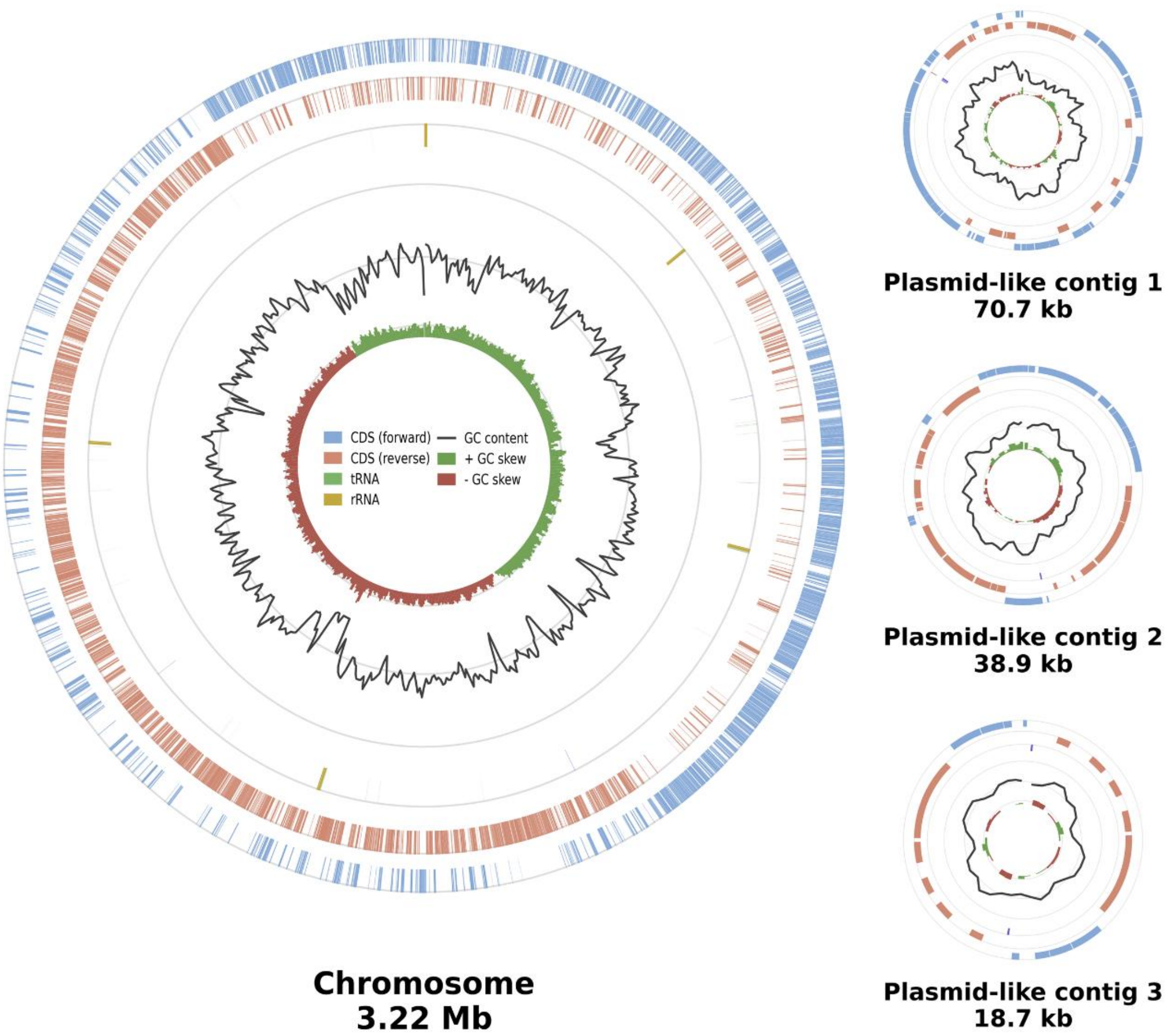

Fig. S4. Circular maps of the chromosome and plasmid-like contigs of *Lactiplantibacillus plantarum* J2K-1229.

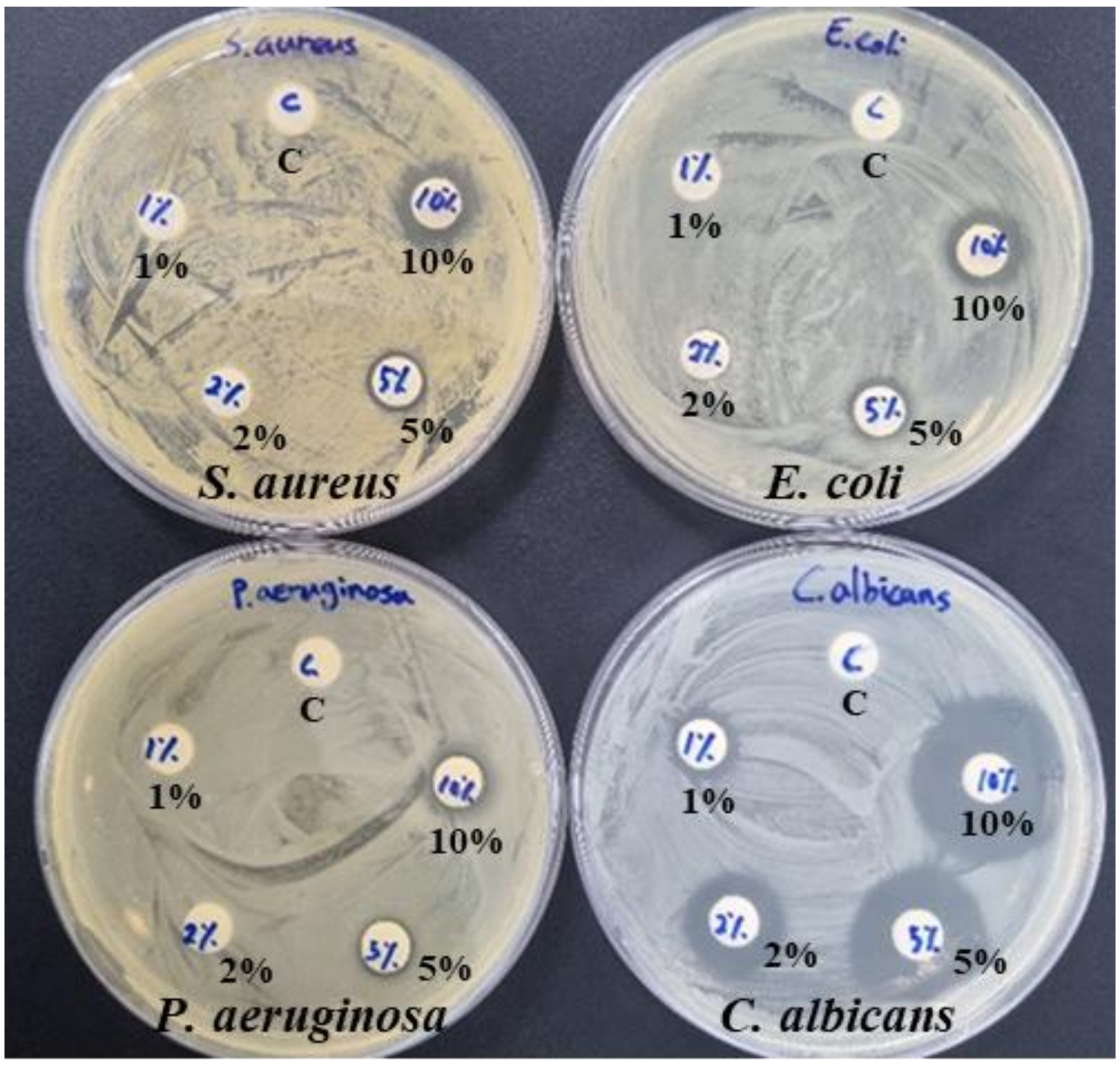

Fig. S5. Disk diffusion test of different concentrations of the purified biosurfactant fraction produced by strain J2K-1229 against different strains: *S. aureus, E. coli*, *P. aeruginosa*, and *C. albicans*. The percentages in the individual discs of the individual plate (1, 2, 5, 10%) represent different concentrations of purified biosurfactants. C, Negative control.

(A)

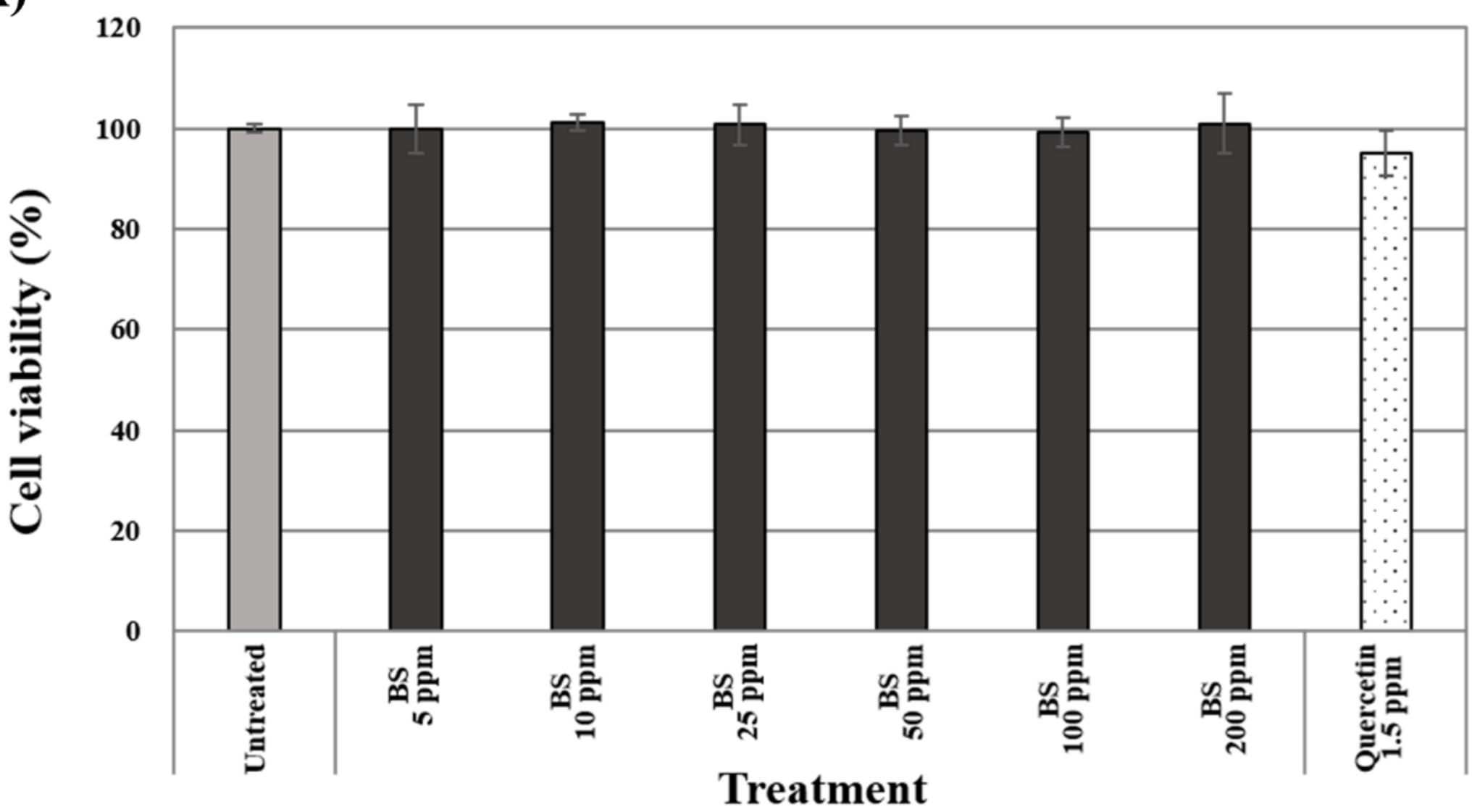

(B)

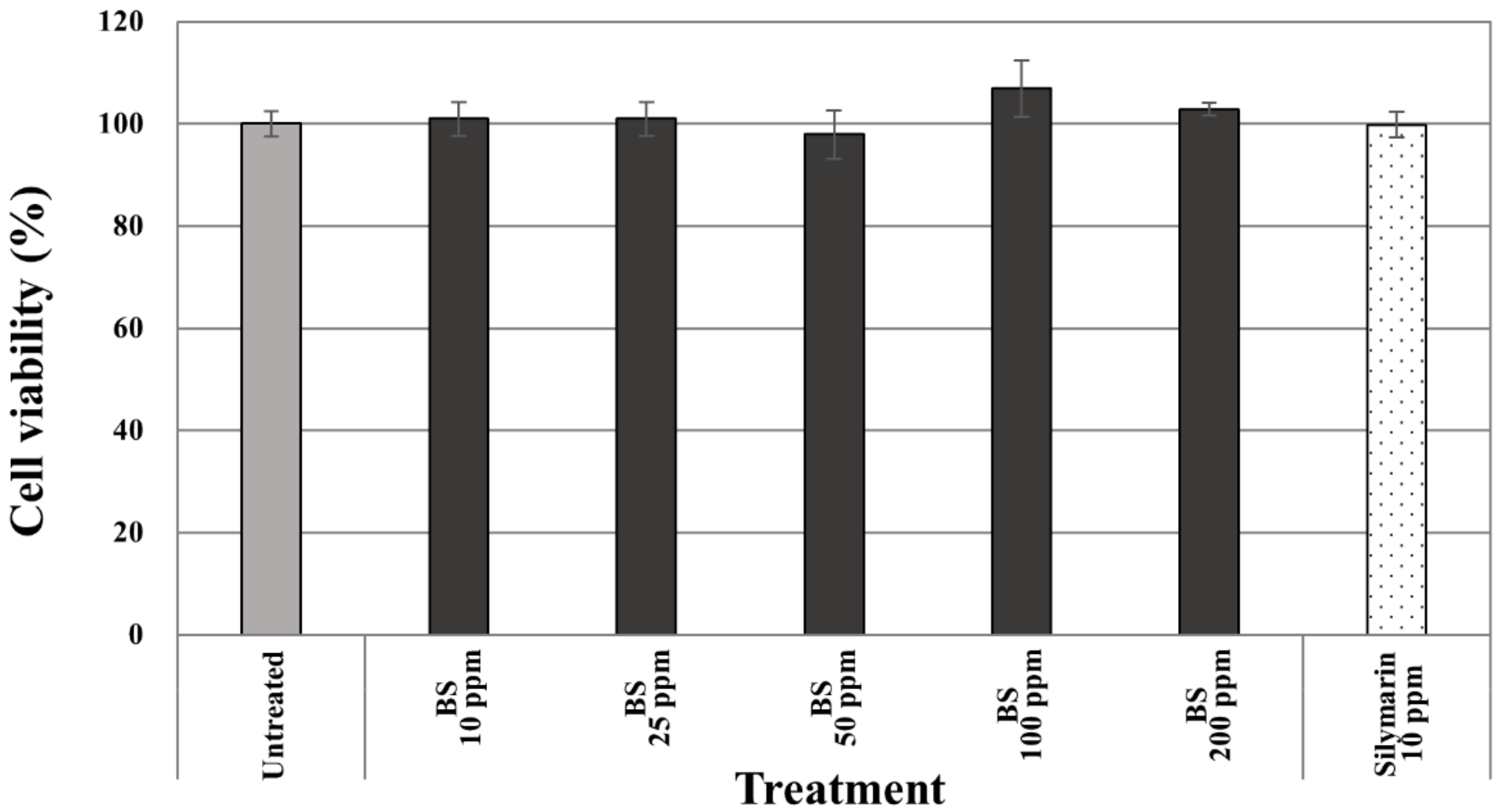

Fig. S6. Effects of the biosurfactant solution (BS solution) on cell viability. (A) Viability of RAW 264.7 macrophages after treatment with different concentrations of the BS solution. **(B)** Viability of HaCaT keratinocytes after treatment with different concentrations of the BS solution. Cell viability was determined by MTT assay after 24 h of treatment. Values are expressed as percentage of the untreated control and presented as mean ± SD (n = 3).
